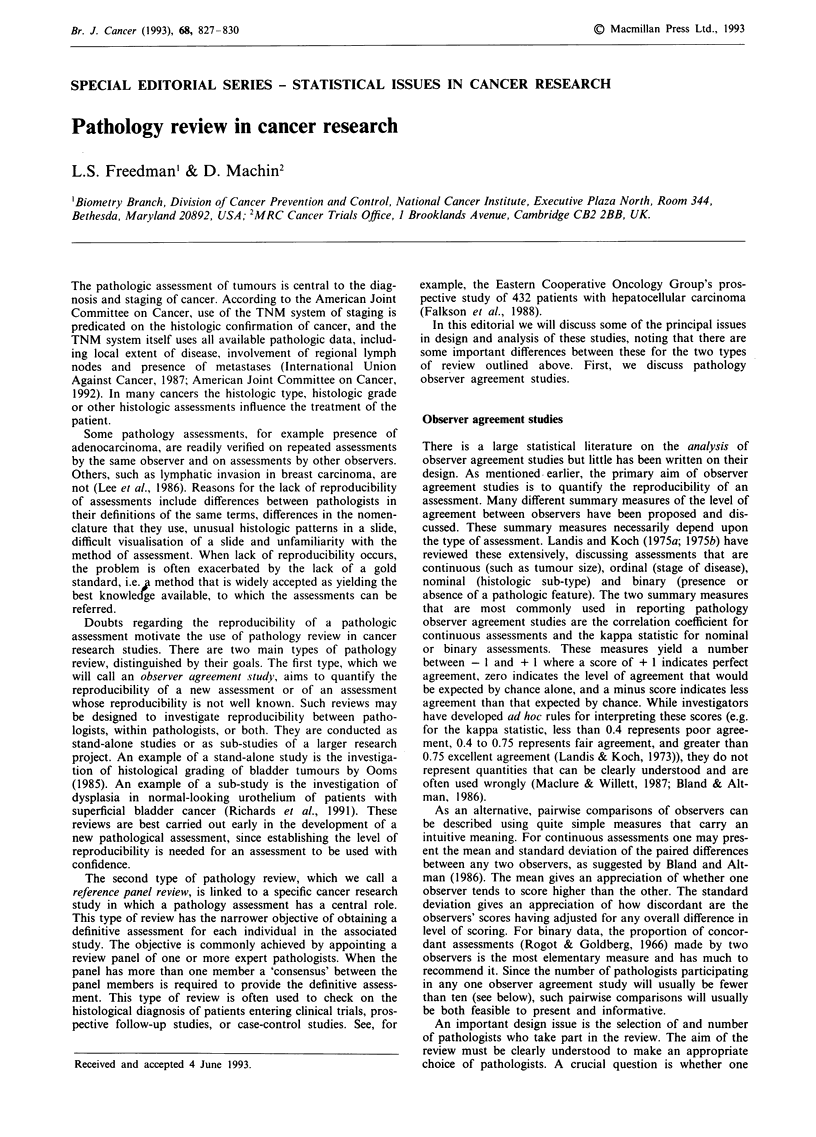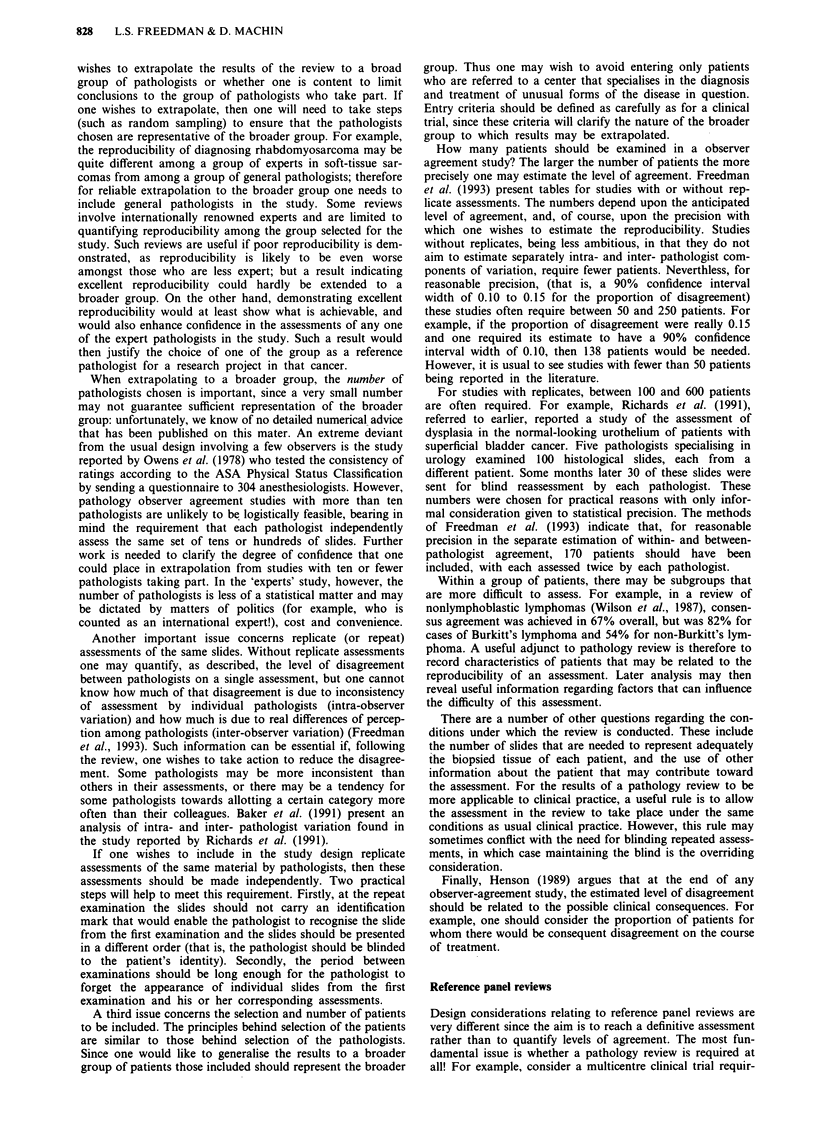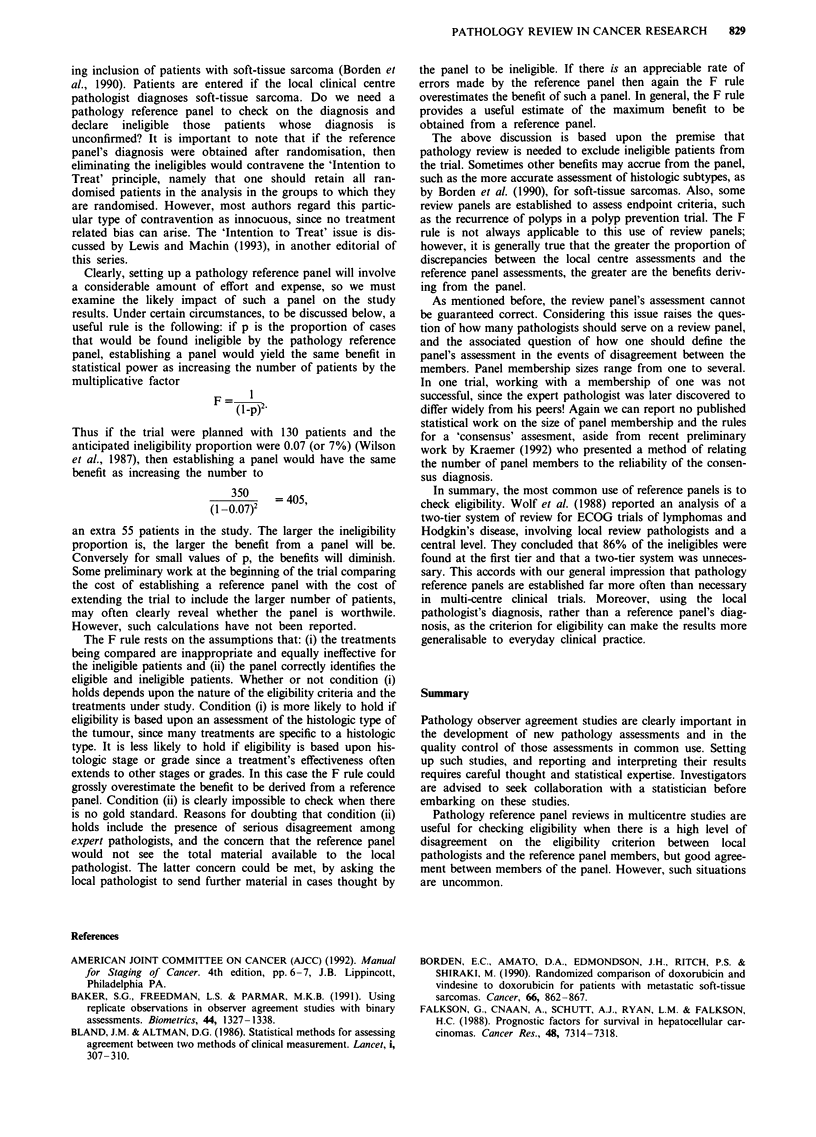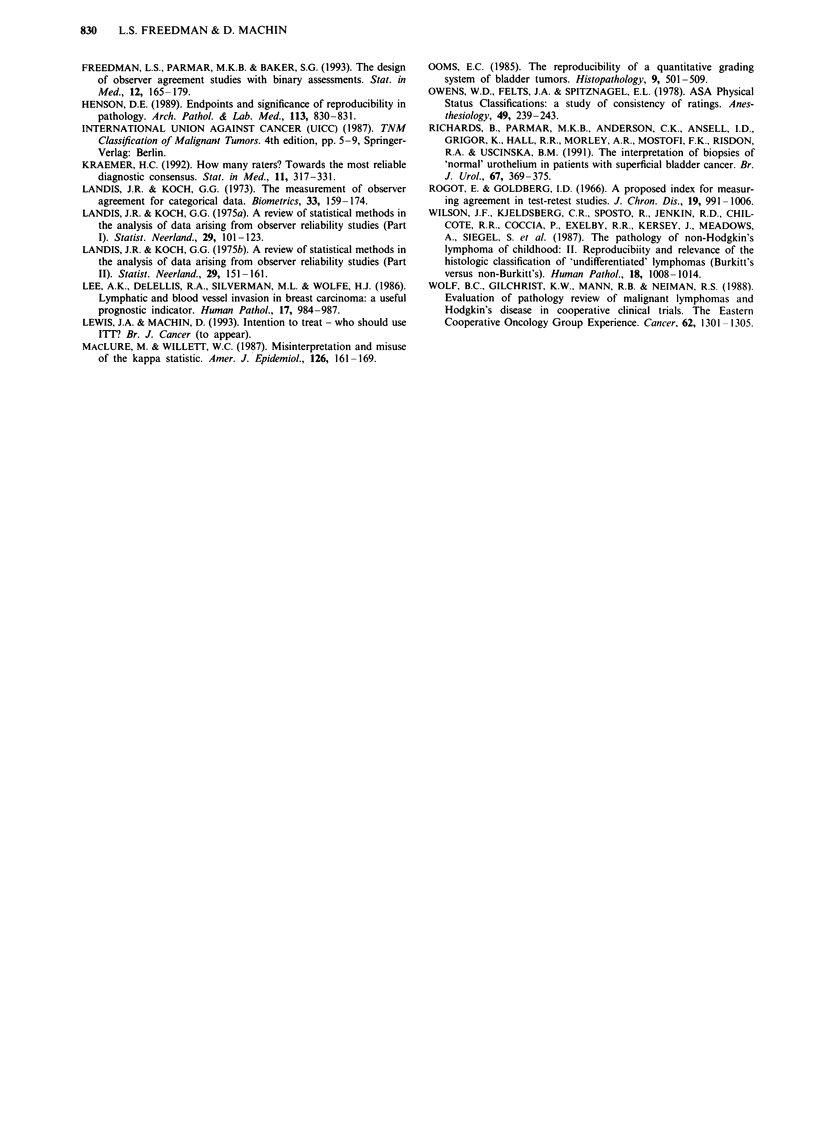# Pathology review in cancer research.

**DOI:** 10.1038/bjc.1993.440

**Published:** 1993-11

**Authors:** L. S. Freedman, D. Machin

## Abstract

Pathology observer agreement studies are clearly important in the development of new pathology assessments and in the quality control of those assessments in common use. Setting up such studies, and reporting and interpreting their results requires careful thought and statistical expertise. Investigators are advised to seek collaboration with a statistician before embarking on these studies. Pathology reference panel reviews in multicentre studies are useful for checking eligibility when there is a high level of disagreement on the eligibility criterion between local pathologists and the reference panel members, but good agreement between members of the panel. However, such situations are uncommon.


Br. J. Cancer (1993), 68, 827-830 © Macmillan Press Ltd., 1993

SPECIAL EDITORIAL SERIES - STATISTICAL ISSUES IN CANCER RESEARCH

### Pathology review in cancer research

L.S. Freedman[1] & D. Machin[2]

[1]*Biometry Branch, Division of Cancer Prevention and Control, National Cancer Institute, Executive Plaza North, Room 344, Bethesda, Maryland 20892, USA;* [2]*MRC Cancer Trials Office, 1 Brooklands Avenue, Cambridge CB2 2BB, UK.*

The pathologic assessment of tumours is central to the diagnosis and staging of cancer. According to the American Joint Committee on Cancer, use of the TNM system of staging is predicated on the histologic confirmation of cancer, and the TNM system itself uses all available pathologic data, including local extent of disease, involvement of regional lymph nodes and presence of metastases (International Union Against Cancer, 1987; American Joint Committee on Cancer, 1992). In many cancers the histologic type, histologic grade or other histologic assessments influence the treatment of the patient.

Some pathology assessments, for example presence of adenocarcinoma, are readily verified on repeated assessments by the same observer and on assessments by other observers. Others, such as lymphatic invasion in breast carcinoma, are not (Lee *et al*., 1986). Reasons for the lack of reproducibility of assessments include differences between pathologists in their definitions of the same terms, differences in the nomenclature that they use, unusual histologic patterns in a slide, difficult visualisation of a slide and unfamiliarity with the method of assessment. When lack of reproducibility occurs, the problem is often exacerbated by the lack of a gold standard, i.e. a method that is widely accepted as yielding the best knowledge available, to which the assessments can be referred.

Doubts regarding the reproducibility of a pathologic assessment motivate the use of pathology review in cancer research studies. There are two main types of pathology review, distinguished by their goals. The first type, which we will call an *observer agreement study*, aims to quantify the reproducibility of a new assessment or of an assessment whose reproducibility is not well known. Such reviews may be designed to investigate reproducibility between pathologists, within pathologists, or both. They are conducted as stand-alone studies or as sub-studies of a larger research project. An example of a stand-alone study is the investigation of histological grading of bladder tumours by Ooms (1985). An example of a sub-study is the investigation of dysplasia in normal-looking urothelium of patients with superficial bladder cancer (Richards *et al*., 1991). These reviews are best carried out early in the development of a new pathological assessment, since establishing the level of reproducibility is needed for an assessment to be used with confidence.

The second type of pathology review, which we call a *reference panel review*, is linked to a specific cancer research study in which a pathology assessment has a central role. This type of review has the narrower objective of obtaining a definitive assessment for each individual in the associated study. The objective is commonly achieved by appointing a review panel of one or more expert pathologists. When the panel has more than one member a 'consensus' between the panel members is required to provide the definitive assessment. This type of review is often used to check on the histological diagnosis of patients entering clinical trials, prospective follow-up studies, or case-control studies. See, for example, the Eastern Cooperative Oncology Group's prospective study of 432 patients with hepatocellular carcinoma (Falkson *et al*., 1988).

In this editorial we will discuss some of the principal issues in design and analysis of these studies, noting that there are some important differences between these for the two types of review outlined above. First, we discuss pathology observer agreement studies.

### Observer agreement studies

There is a large statistical literature on the *analysis* of observer agreement studies but little has been written on their design. As mentioned earlier, the primary aim of observer agreement studies is to quantify the reproducibility of an assessment. Many different summary measures of the level of agreement between observers have been proposed and discussed. These summary measures necessarily depend upon the type of assessment. Landis and Koch (1975*a*; 1975*b*) have reviewed these extensively, discussing assessments that are continuous (such as tumour size), ordinal (stage of disease), nominal (histologic sub-type) and binary (presence or absence of a pathologic feature). The two summary measures that are most commonly used in reporting pathology observer agreement studies are the correlation coefficient for continuous assessments and the kappa statistic for nominal or binary assessments. These measures yield a number between − 1 and + 1 where a score of + 1 indicates perfect agreement, zero indicates the level of agreement that would be expected by chance alone, and a minus score indicates less agreement than that expected by chance. While investigators have developed *ad hoc* rules for interpreting these scores (e.g. for the kappa statistic, less than 0.4 represents poor agreement, 0.4 to 0.75 represents fair agreement, and greater than 0.75 excellent agreement (Landis & Koch, 1973)), they do not represent quantities that can be clearly understood and are often used wrongly (Maclure & Willett, 1987; Bland & Altman, 1986).

As an alternative, pairwise comparisons of observers can be described using quite simple measures that carry an intuitive meaning. For continuous assessments one may present the mean and standard deviation of the paired differences between any two observers, as suggested by Bland and Altman (1986). The mean gives an appreciation of whether one observer tends to score higher than the other. The standard deviation gives an appreciation of how discordant are the observers' scores having adjusted for any overall difference in level of scoring. For binary data, the proportion of concordant assessments (Rogot & Goldberg, 1966) made by two observers is the most elementary measure and has much to recommend it. Since the number of pathologists participating in any one observer agreement study will usually be fewer than ten (see below), such pairwise comparisons will usually be both feasible to present and informative.

An important design issue is the selection of and number of pathologists who take part in the review. The aim of the review must be clearly understood to make an appropriate choice of pathologists. A crucial question is whether one

Received and accepted 4 June 1993.

wishes to extrapolate the results of the review to a broad group of pathologists or whether one is content to limit conclusions to the group of pathologists who take part. If one wishes to extrapolate, then one will need to take steps (such as random sampling) to ensure that the pathologists chosen are representative of the broader group. For example, the reproducibility of diagnosing rhabdomyosarcoma may be quite different among a group of experts in soft-tissue sarcomas from among a group of general pathologists; therefore for reliable extrapolation to the broader group one needs to include general pathologists in the study. Some reviews involve internationally renowned experts and are limited to quantifying reproducibility among the group selected for the study. Such reviews are useful if poor reproducibility is demonstrated, as reproducibility is likely to be even worse amongst those who are less expert; but a result indicating excellent reproducibility could hardly be extended to a broader group. On the other hand, demonstrating excellent reproducibility would at least show what is achievable, and would also enhance confidence in the assessments of any one of the expert pathologists in the study. Such a result would then justify the choice of one of the group as a reference pathologist for a research project in that cancer.

When extrapolating to a broader group, the *number* of pathologists chosen is important, since a very small number may not guarantee sufficient representation of the broader group: unfortunately, we know of no detailed numerical advice that has been published on this mater. An extreme deviant from the usual design involving a few observers is the study reported by Owens *et al.* (1978) who tested the consistency of ratings according to the ASA Physical Status Classification by sending a questionnaire to 304 anesthesiologists. However, pathology observer agreement studies with more than ten pathologists are unlikely to be logistically feasible, bearing in mind the requirement that each pathologist independently assess the same set of tens or hundreds of slides. Further work is needed to clarify the degree of confidence that one could place in extrapolation from studies with ten or fewer pathologists taking part. In the 'experts' study, however, the number of pathologists is less of a statistical matter and may be dictated by matters of politics (for example, who is counted as an international expert!), cost and convenience.

Another important issue concerns replicate (or repeat) assessments of the same slides. Without replicate assessments one may quantify, as described, the level of disagreement between pathologists on a single assessment, but one cannot know how much of that disagreement is due to inconsistency of assessment by individual pathologists (intra-observer variation) and how much is due to real differences of perception among pathologists (inter-observer variation) (Freedman *et al.*, 1993). Such information can be essential if, following the review, one wishes to take action to reduce the disagreement. Some pathologists may be more inconsistent than others in their assessments, or there may be a tendency for some pathologists towards allotting a certain category more often than their colleagues. Baker *et al.* (1991) present an analysis of intra- and inter- pathologist variation found in the study reported by Richards *et al.* (1991).

If one wishes to include in the study design replicate assessments of the same material by pathologists, then these assessments should be made independently. Two practical steps will help to meet this requirement. Firstly, at the repeat examination the slides should not carry an identification mark that would enable the pathologist to recognise the slide from the first examination and the slides should be presented in a different order (that is, the pathologist should be blinded to the patient's identity). Secondly, the period between examinations should be long enough for the pathologist to forget the appearance of individual slides from the first examination and his or her corresponding assessments.

A third issue concerns the selection and number of patients to be included. The principles behind selection of the patients are similar to those behind selection of the pathologists. Since one would like to generalise the results to a broader group of patients those included should represent the broader group. Thus one may wish to avoid entering only patients who are referred to a center that specialises in the diagnosis and treatment of unusual forms of the disease in question. Entry criteria should be defined as carefully as for a clinical trial, since these criteria will clarify the nature of the broader group to which results may be extrapolated.

How many patients should be examined in a observer agreement study? The larger the number of patients the more precisely one may estimate the level of agreement. Freedman *et al.* (1993) present tables for studies with or without replicate assessments. The numbers depend upon the anticipated level of agreement, and, of course, upon the precision with which one wishes to estimate the reproducibility. Studies without replicates, being less ambitious, in that they do not aim to estimate separately intra- and inter- pathologist components of variation, require fewer patients. Neverthless, for reasonable precision, (that is, a 90% confidence interval width of 0.10 to 0.15 for the proportion of disagreement) these studies often require between 50 and 250 patients. For example, if the proportion of disagreement were really 0.15 and one required its estimate to have a 90% confidence interval width of 0.10, then 138 patients would be needed. However, it is usual to see studies with fewer than 50 patients being reported in the literature.

For studies with replicates, between 100 and 600 patients are often required. For example, Richards *et al.* (1991), referred to earlier, reported a study of the assessment of dysplasia in the normal-looking urothelium of patients with superficial bladder cancer. Five pathologists specialising in urology examined 100 histological slides, each from a different patient. Some months later 30 of these slides were sent for blind reassessment by each pathologist. These numbers were chosen for practical reasons with only informal consideration given to statistical precision. The methods of Freedman *et al.* (1993) indicate that, for reasonable precision in the separate estimation of within- and between-pathologist agreement, 170 patients should have been included, with each assessed twice by each pathologist.

Within a group of patients, there may be subgroups that are more difficult to assess. For example, in a review of nonlymphoblastic lymphomas (Wilson *et al.*, 1987), consensus agreement was achieved in 67% overall, but was 82% for cases of Burkitt's lymphoma and 54% for non-Burkitt's lymphoma. A useful adjunct to pathology review is therefore to record characteristics of patients that may be related to the reproducibility of an assessment. Later analysis may then reveal useful information regarding factors that can influence the difficulty of this assessment.

There are a number of other questions regarding the conditions under which the review is conducted. These include the number of slides that are needed to represent adequately the biopsied tissue of each patient, and the use of other information about the patient that may contribute toward the assessment. For the results of a pathology review to be more applicable to clinical practice, a useful rule is to allow the assessment in the review to take place under the same conditions as usual clinical practice. However, this rule may sometimes conflict with the need for blinding repeated assessments, in which case maintaining the blind is the overriding consideration.

Finally, Henson (1989) argues that at the end of any observer-agreement study, the estimated level of disagreement should be related to the possible clinical consequences. For example, one should consider the proportion of patients for whom there would be consequent disagreement on the course of treatment.

**Reference panel reviews**

Design considerations relating to reference panel reviews are very different since the aim is to reach a definitive assessment rather than to quantify levels of agreement. The most fundamental issue is whether a pathology review is required at all! For example, consider a multicentre clinical trial requiring inclusion of patients with soft-tissue sarcoma (Borden *et al.*, 1990). Patients are entered if the local clinical centre pathologist diagnoses soft-tissue sarcoma. Do we need a pathology reference panel to check on the diagnosis and declare ineligible those patients whose diagnosis is unconfirmed? It is important to note that if the reference panel's diagnosis were obtained after randomisation, then eliminating the ineligibles would contravene the 'Intention to Treat' principle, namely that one should retain all randomised patients in the analysis in the groups to which they are randomised. However, most authors regard this particular type of contravention as innocuous, since no treatment related bias can arise. The 'Intention to Treat' issue is discussed by Lewis and Machin (1993), in another editorial of this series.

Clearly, setting up a pathology reference panel will involve a considerable amount of effort and expense, so we must examine the likely impact of such a panel on the study results. Under certain circumstances, to be discussed below, a useful rule is the following: if p is the proportion of cases that would be found ineligible by the pathology reference panel, establishing a panel would yield the same benefit in statistical power as increasing the number of patients by the multiplicative factor

$$F = \frac{1}{(1-p)^2}.$$

Thus if the trial were planned with 130 patients and the anticipated ineligibility proportion were 0.07 (or 7%) (Wilson *et al.*, 1987), then establishing a panel would have the same benefit as increasing the number to

$$\frac{350}{(1-0.07)^2} = 405,$$

an extra 55 patients in the study. The larger the ineligibility proportion is, the larger the benefit from a panel will be. Conversely for small values of p, the benefits will diminish. Some preliminary work at the beginning of the trial comparing the cost of establishing a reference panel with the cost of extending the trial to include the larger number of patients, may often clearly reveal whether the panel is worthwile. However, such calculations have not been reported.

The F rule rests on the assumptions that: (i) the treatments being compared are inappropriate and equally ineffective for the ineligible patients and (ii) the panel correctly identifies the eligible and ineligible patients. Whether or not condition (i) holds depends upon the nature of the eligibility criteria and the treatments under study. Condition (i) is more likely to hold if eligibility is based upon an assessment of the histologic type of the tumour, since many treatments are specific to a histologic type. It is less likely to hold if eligibility is based upon histologic stage or grade since a treatment's effectiveness often extends to other stages or grades. In this case the F rule could grossly overestimate the benefit to be derived from a reference panel. Condition (ii) is clearly impossible to check when there is no gold standard. Reasons for doubting that condition (ii) holds include the presence of serious disagreement among *expert* pathologists, and the concern that the reference panel would not see the total material available to the local pathologist. The latter concern could be met, by asking the local pathologist to send further material in cases thought by the panel to be ineligible. If there *is* an appreciable rate of errors made by the reference panel then again the F rule overestimates the benefit of such a panel. In general, the F rule provides a useful estimate of the maximum benefit to be obtained from a reference panel.

The above discussion is based upon the premise that pathology review is needed to exclude ineligible patients from the trial. Sometimes other benefits may accrue from the panel, such as the more accurate assessment of histologic subtypes, as by Borden *et al.* (1990), for soft-tissue sarcomas. Also, some review panels are established to assess endpoint criteria, such as the recurrence of polyps in a polyp prevention trial. The F rule is not always applicable to this use of review panels; however, it is generally true that the greater the proportion of discrepancies between the local centre assessments and the reference panel assessments, the greater are the benefits deriving from the panel.

As mentioned before, the review panel's assessment cannot be guaranteed correct. Considering this issue raises the question of how many pathologists should serve on a review panel, and the associated question of how one should define the panel's assessment in the events of disagreement between the members. Panel membership sizes range from one to several. In one trial, working with a membership of one was not successful, since the expert pathologist was later discovered to differ widely from his peers! Again we can report no published statistical work on the size of panel membership and the rules for a 'consensus' assesment, aside from recent preliminary work by Kraemer (1992) who presented a method of relating the number of panel members to the reliability of the consensus diagnosis.

In summary, the most common use of reference panels is to check eligibility. Wolf *et al.* (1988) reported an analysis of a two-tier system of review for ECOG trials of lymphomas and Hodgkin's disease, involving local review pathologists and a central level. They concluded that 86% of the ineligibles were found at the first tier and that a two-tier system was unnecessary. This accords with our general impression that pathology reference panels are established far more often than necessary in multi-centre clinical trials. Moreover, using the local pathologist's diagnosis, rather than a reference panel's diagnosis, as the criterion for eligibility can make the results more generalisable to everyday clinical practice.

### Summary

Pathology observer agreement studies are clearly important in the development of new pathology assessments and in the quality control of those assessments in common use. Setting up such studies, and reporting and interpreting their results requires careful thought and statistical expertise. Investigators are advised to seek collaboration with a statistician before embarking on these studies.

Pathology reference panel reviews in multicentre studies are useful for checking eligibility when there is a high level of disagreement on the eligibility criterion between local pathologists and the reference panel members, but good agreement between members of the panel. However, such situations are uncommon.

### References

AMERICAN JOINT COMMITTEE ON CANCER (AJCC) (1992). *Manual for Staging of Cancer*. 4th edition, pp. 6–7, J.B. Lippincott, Philadelphia PA.

BAKER, S.G., FREEDMAN, L.S. & PARMAR, M.K.B. (1991). Using replicate observations in observer agreement studies with binary assessments. *Biometrics*, **44**, 1327–1338.

BLAND, J.M. & ALTMAN, D.G. (1986). Statistical methods for assessing agreement between two methods of clinical measurement. *Lancet*, **i**, 307–310.

BORDEN, E.C., AMATO, D.A., EDMONDSON, J.H., RITCH, P.S. & SHIRAKI, M. (1990). Randomized comparison of doxorubicin and vindesine to doxorubicin for patients with metastatic soft-tissue sarcomas. *Cancer*, **66**, 862–867.

FALKSON, G., CNAAN, A., SCHUTT, A.J., RYAN, L.M. & FALKSON, H.C. (1988). Prognostic factors for survival in hepatocellular carcinomas. *Cancer Res.*, **48**, 7314–7318.

FREEDMAN, L.S., PARMAR, M.K.B. & BAKER, S.G. (1993). The design of observer agreement studies with binary assessments. *Stat. in Med.*, **12**, 165–179.

HENSON, D.E. (1989). Endpoints and significance of reproducibility in pathology. *Arch. Pathol. & Lab. Med.*, **113**, 830-831.

INTERNATIONAL UNION AGAINST CANCER (UICC) (1987). *TNM Classification of Malignant Tumors*. 4th edition, pp. 5–9, Springer-Verlag: Berlin.

KRAEMER, H.C. (1992). How many raters? Towards the most reliable diagnostic consensus. *Stat. in Med.*, **11**, 317–331.

LANDIS, J.R. & KOCH, G.G. (1973). The measurement of observer agreement for categorical data. *Biometrics*, **33**, 159–174.

LANDIS, J.R. & KOCH, G.G. (1975*a*). A review of statistical methods in the analysis of data arising from observer reliability studies (Part I). *Statist. Neerland.*, **29**, 101–123.

LANDIS, J.R. & KOCH, G.G. (1975*b*). A review of statistical methods in the analysis of data arising from observer reliability studies (Part II). *Statist. Neerland.*, **29**, 151–161.

LEE, A.K., DELELLIS, R.A., SILVERMAN, M.L. & WOLFE, H.J. (1986). Lymphatic and blood vessel invasion in breast carcinoma: a useful prognostic indicator. *Human Pathol.*, **17**, 984-987.

LEWIS, J.A. & MACHIN, D. (1993). Intention to treat – who should use ITT? *Br. J. Cancer* (to appear).

MACLURE, M. & WILLETT, W.C. (1987). Misinterpretation and misuse of the kappa statistic. *Amer. J. Epidemiol.*, **126**, 161–169.

OOMS, E.C. (1985). The reproducibility of a quantitative grading system of bladder tumors. *Histopathology*, **9**, 501–509.

OWENS, W.D., FELTS, J.A. & SPITZNAGEL, E.L. (1978). ASA Physical Status Classifications: a study of consistency of ratings. *Anesthesiology*, **49**, 239–243.

RICHARDS, B., PARMAR, M.K.B., ANDERSON, C.K., ANSELL, I.D., GRIGOR, K., HALL, R.R., MORLEY, A.R., MOSTOFI, F.K., RISDON, R.A. & USCINSKA, B.M. (1991). The interpretation of biopsies of 'normal' urothelium in patients with superficial bladder cancer. *Br. J. Urol.*, **67**, 369–375.

ROGOT, E. & GOLDBERG, I.D. (1966). A proposed index for measuring agreement in test-retest studies. *J. Chron. Dis.*, **19**, 991–1006.

WILSON, J.F., KJELDSBERG, C.R., SPOSTO, R., JENKIN, R.D., CHILCOTE, R.R., COCCIA, P., EXELBY, R.R., KERSEY, J., MEADOWS, A., SIEGEL, S. *et al.* (1987). The pathology of non-Hodgkin's lymphoma of childhood: II. Reproducibiity and relevance of the histologic classification of 'undifferentiated' lymphomas (Burkitt's versus non-Burkitt's). *Human Pathol.*, **18**, 1008–1014.

WOLF, B.C., GILCHRIST, K.W., MANN, R.B. & NEIMAN, R.S. (1988). Evaluation of pathology review of malignant lymphomas and Hodgkin's disease in cooperative clinical trials. The Eastern Cooperative Oncology Group Experience. *Cancer*, **62**, 1301–1305.